# Anomalous correlation effects and unique phase diagram of electron-doped FeSe revealed by photoemission spectroscopy

**DOI:** 10.1038/ncomms10840

**Published:** 2016-03-08

**Authors:** C. H. P. Wen, H. C. Xu, C. Chen, Z. C. Huang, X. Lou, Y. J. Pu, Q. Song, B. P. Xie, Mahmoud Abdel-Hafiez, D. A. Chareev, A. N. Vasiliev, R. Peng, D. L. Feng

**Affiliations:** 1 State Key Laboratory of Surface Physics, Department of Physics and Advanced Materials Laboratory, Fudan University, Shanghai 200433, China; 2 Institute of Physics, Goethe University Frankfurt, 60438 Frankfurt, Germany; 3 Center for High Pressure Science and Technology Advanced Research, 1690 Cailun Road, Shanghai 201203, China; 4 Institute of Experimental Mineralogy, Russian Academy of Sciences, Chernogolovka, 119991 Moscow , Russia; 5 Low Temperature Physics and Superconductivity Department, M.V. Lomonosov Moscow State University, 119991 Moscow, Russia

## Abstract

FeSe layer-based superconductors exhibit exotic and distinctive properties. The undoped FeSe shows nematicity and superconductivity, while the heavily electron-doped K_*x*_Fe_2−*y*_Se_2_ and single-layer FeSe/SrTiO_3_ possess high superconducting transition temperatures that pose theoretical challenges. However, a comprehensive study on the doping dependence of an FeSe layer-based superconductor is still lacking due to the lack of a clean means of doping control. Through angle-resolved photoemission spectroscopy studies on K-dosed thick FeSe films and FeSe_0.93_S_0.07_ bulk crystals, here we reveal the internal connections between these two types of FeSe-based superconductors, and obtain superconductivity below ∼46 K in an FeSe layer under electron doping without interfacial effects. Moreover, we discover an exotic phase diagram of FeSe with electron doping, including a nematic phase, a superconducting dome, a correlation-driven insulating phase and a metallic phase. Such an anomalous phase diagram unveils the remarkable complexity, and highlights the importance of correlations in FeSe layer-based superconductors.

## Abstract (web summary)

Electron doping is a powerful way to induce quantum phase transitions in materials and explore exotic states of matter. Here, Wen *et al.* present carefully-controlled potassium dosing in FeSe films and FeSe_0.93_S_0.07_ bulk, which enhances superconductivity and induces other anomalous phases, revealing a complex phase diagram.

## Graphical abstract

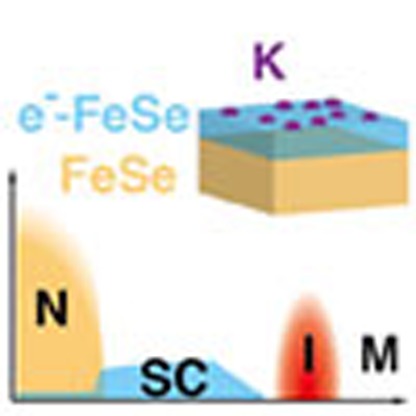

Carrier doping is a critical parameter that governs the electronic correlations and ground states in high-temperature superconductors. Extending a superconducting system to an unexplored doping regime often deepens our understanding of its mechanism. One example is the insights brought by the discovery of heavily electron-doped FeSe layer-based superconductors^1^,^2^,^3^,^4^,^5^,^6^,^7^,^8^,^9^. Compared with undoped FeSe, the enhanced superconductivity in heavily electron-doped FeSe superconductors without any hole Fermi surface^5^,^6^,^7^,^8^,^9^,^10^,^11^ challenges the prevailing pairing picture based on the nesting between electron and hole Fermi surfaces. Moreover, unlike the moderate correlation strength in most iron pnictides, it is reported that the heavily electron-doped FeSe is strongly correlated and near a Mott insulating phase^12^,^13^, suggesting that the underlying physics may be unified with the cuprate superconductors. To bridge the knowledge gap between these systems, it is crucial to figure out how the superconductivity and correlation behaviour evolve with doping by constructing an FeSe layer-based system with clean and systematic doping control.

Systematic control of the electron doping in a pure iron selenide superconductor is still lacking. Although heavy electron doping has been achieved in intercalated FeSe crystals such as *A*_*x*_Fe_2−*y*_Se_2_ (*A*=K, Rb, Cs and Tl/K)^1^,^2^ and (Li_0.8_Fe_0.2_)OHFeSe (ref. 3), the doping levels are discrete and fixed. Moreover, microscopic phase separation in *A*_*x*_Fe_2−*y*_Se_2_ (refs 12, 14, 15, 16, 17, 18, 19, 20) complicates studies of the intrinsic superconductivity. In (Li_0.8_Fe_0.2_)OHFeSe, the polar surface prevents the observation of intrinsic bulk electronic structure in surface sensitive angle-resolved photoemission spectroscopy (ARPES) measurements^11^. In single-layer FeSe films on SrTiO_3_ or BaTiO_3_, heavy electron doping is induced by charge transfer from the oxygen-deficient substrate^6^, which is difficult to control reliably. It has been reported that post annealing in vacuum can vary the doping in single-layer FeSe films on SrTiO_3_ substrates^7^,^21^; however, this approach could also vary the stoichiometry and morphology of the FeSe films^7^,^21^,^22^, and also fails to induce superconductivity in the second FeSe layer^22^. Moreover, interfacial effects have been suggested to be crucial for the enhanced superconductivity^9^,^23^, which further complicates the issue. Recently, by controlling the doping via K dosing, a superconducting dome has been observed in FeSe films of 3-uc (unit cell) thickness^24^. However, no superconductivity was found in 20-uc FeSe films down to 13 K at any doping^24^, so the enhanced superconductivity in 3-uc FeSe/SrTiO_3_ was attributed to certain interfacial effect^24^.

Here we report systematic ARPES studies on the electron-doping-induced effects in both thick FeSe films up to 50 uc and FeSe_0.93_S_0.07_ bulk crystals via K dosing. With increased doping, the nematic order is suppressed, while the superconductivity is enhanced from a low superconducting transition temperature (*T*_c_) FeSe system with both electron and hole Fermi surfaces to a high *T*_c_ (up to 46 K) heavily electron-doped FeSe system with electron Fermi surfaces only. Remarkably, the correlation strength of the system is enhanced with increased doping, opposite to what usually happens in iron pnictides, such as NaFe_1−*x*_Co_*x*_As and LiFe_1−*x*_Co_*x*_As (ref. 25). Consequently, there is a superconductor-to-insulator transition driven by the correlations. Finally, a metallic phase appears in the far overdoped regime. Our results provide the most comprehensive phase diagram of FeSe with electron doping in a clean system, demonstrating that it is exotic and distinct from those of other Fe-based superconductors. In addition to extending the phase diagram of electron-doped FeSe into two unexplored phases, our findings offer a foundation for the global understanding of the interplay among nematic order, superconductivity and electron correlations in the FeSe layer-based superconductors.

## Results

### Electron doping and enhanced superconductivity

**Figure 1:**
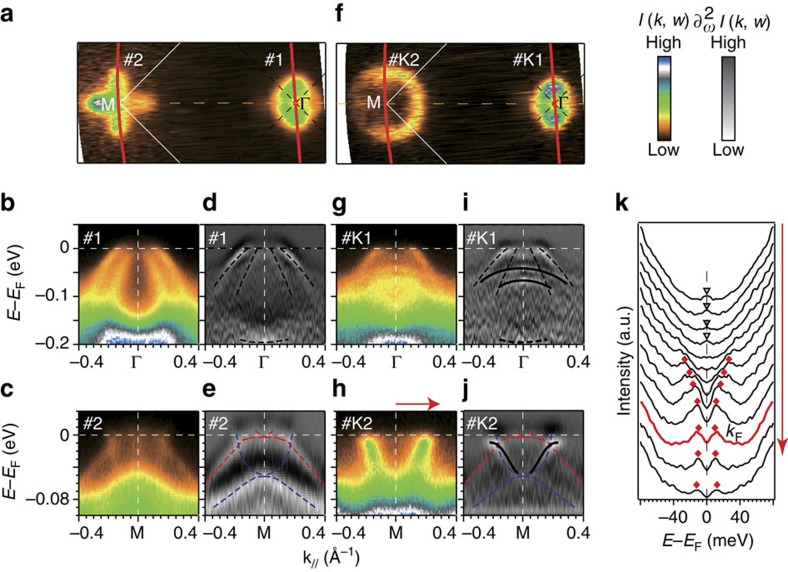
Electronic structures before and after K dosing for a 30  uc FeSe film on SrTiO_3_. (**a**) Photoemission intensity map at the Fermi energy (*E*_F_) for a 30 uc FeSe film. The intensity was integrated over an energy window of (*E*_F_−10 meV, *E*_F_+10 meV). The red curves indicate the momentum locations of the cuts #1 and #2. (**b**,**d**) Photoemission intensity along cut #1 in **a** and the corresponding second derivative, respectively. (**c**,**e**) The same as **b**,**d** but along cut #2 in **a**. (**f**) Photoemission intensity map over an energy window of (*E*_F_−10 meV, *E*_F_+10 meV) for the 30 uc FeSe film with electron doping *x*=0.098 after K dosing. The red curves indicate the momentum locations of the cuts #K1 and #K2. (**g**,**i**) Photoemission intensity along cut #K1 in **f** and the corresponding second derivative, respectively. (**h**,**j**) The same as **g**,**i** but along cut #K2 in **f**. (**k**) Symmetrized energy distribution curves along the momenta indicated by the arrows in **h**. The data in **h**,**j** and **k** were taken at 31 K, the others at 70 K.

Figure 1 shows the band structures of a 30-uc thick FeSe film before and after K dosing. Before K dosing, the band structure of the 30-uc FeSe film is consistent with those in previous reports on thick FeSe films^6^,^26^ and bulk FeSe crystals^27^,^28^,^29^,^30^. The Fermi surfaces consist of hole pockets at Γ (Fig. 1a), contributed by the two hole-like bands crossing *E*_F_ around Γ (Fig. 1b,d). Around M, there is dumbbell-shaped spectral weight (Fig. 1a) contributed by the complex band structure, which is due to the splitting of bands with d_*xz*_ and d_*yz*_ orbital characters (Fig. 1c,e)^6^,^26^, a hallmark of the orbital ordering or nematicity. After K dosing, which introduces electrons to FeSe, a circular electron pocket appears around M (Fig. 1f). The photoemission spectra show the superposition of two sets of band structures. One set of bands follow the band structure of undoped FeSe and show weaker spectral weight, as indicated by dashed curves in Fig. 1i,j. Considering the finite detection depth of our ARPES measurement^6^, these bands are attributed to the interior FeSe layers that are undoped. The other set of bands with the prominent photoemission spectral weight comes from the topmost layer that is heavily electron doped. Around Γ, the two hole-like bands shift to higher binding energies and become flatter (solid curves in Fig. 1g,i). A simple electron-like band appears around M (solid curves in Fig. 1h,j), indicating that the nematic order is suppressed^6^. Considering that this electronic structure is similar to that in other heavily electron-doped iron chalcogenides, the electronic states near Fermi energy in K-dosed FeSe should as well be contributed by electrons with d_*xz*_, d_*yz*_ and d_*xy*_ orbitals^31^. There is no band structure corresponding to an intermediate doping level, so we conclude that the electron doping induced by K dosing is confined to the topmost single-unit-cell layer of FeSe. Given the quasi-two-dimentional nature of such a single-unit-cell thick layer of FeSe, its band structure should barely disperse along the k_*z*_ direction. Therefore, the Fermi surface volume measured at this photon energy reflects the electron doping in the FeSe layer on the basis of Luttinger volume. The estimated carrier concentration is 0.098 electrons per Fe (*x*=0.098±0.005). Intriguingly, the symmetrized energy distribution curves in Fig. 1k exhibit back bending after passing the Fermi momentum (k_*F*_) without crossing the Fermi energy. The sharp coherence peaks and back-bending behaviour are hallmarks of Bogoliubov quasiparticles, which implies superconductivity in the K-dosed FeSe. The superconducting gap size is about 10 meV at 31 K, suggesting that the *T*_c_ in this layer is significantly enhanced from the bulk *T*_c_ of 8 K (ref. 32). The weak features from the undoped interior layers remain gapless around M (Fig. 1k), indicating that the superconductivity only exists in the doped topmost layer, without extending via proximity effect into the layers beneath. Our results are in contrast to the absence of superconductivity in 35-uc Fe_0.92_Co_0.08_Se thick films^9^. Compared with Fe_0.92_Co_0.08_Se, the noticeably sharper lineshape of the momentum distribution curves (Supplementary Fig. 1) and the enhanced superconductivity in K-dosed FeSe suggest much weaker impurity scattering in FeSe doped by off-FeSe-plane K atoms than the in-FeSe-plane Co ions^25^,^33^. The lower *T*_c_ in Co-doped FeSe suggests the strong pair breaking effect of Co in heavily electron-doped FeSe.

### Absence of interfacial effect

**Figure 2:**
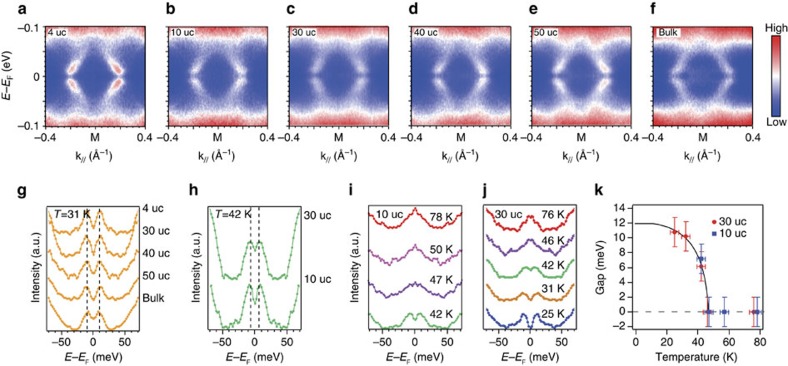
Superconducting gaps in K-dosed FeSe films with varied thicknesses and that of K-dosed FeSe_0.93_S_0.07_ bulk crystals. (**a-f**) Symmetrized photoemission spectra of K-dosed FeSe films with thickness of 4 uc, 10 uc, 30 uc, 40 uc, 50 uc and K-dosed bulk FeSe_0.93_S_0.07_, respectively. The data for 10 uc were taken at 42 K, the others at 31 K. (**g**) Symmetrized energy distribution curves (EDCs) at the Fermi momenta for the FeSe films with different thicknesses and FeSe_0.93_S_0.07_ bulk crystal at 31 K after K dosing. (**h**) The symmetrized EDCs at the Fermi momenta for FeSe films with thicknesses of 10 uc and 30 uc at 42 K after K dosing. (**i**,**j**) Temperature dependences of the symmetrized EDCs at *k*_F_ for thicknesses of 10 uc and 30 uc, respectively. (**k**) Superconducting gap sizes as a function of temperatures from the data in **i**,**j**. The solid curve is the result of a Bardeen–Cooper–Schrieffer formula fit. The doping levels are 0.094±0.005, 0.097±0.005, 0.098±0.005, 0.087±0.005, 0.105±0.005 and 0.115±0.005 electrons per Fe for **a**–**f** respectively. Temperature error bars are due to measurement uncertainties. The error bars of the superconducting gaps are due to the s.d. of the fitting with a typical superconducting-state spectral function.

Figure 2 compares the superconducting gaps of K-dosed FeSe films with various thicknesses and that of K-dosed FeSe_0.93_S_0.07_ bulk crystals (*T*_c_=9.7 K without K dosing^34^). At an electron doping level around *x*=0.09, back-bending dispersions and superconducting gaps are observed for all the K-dosed FeSe films with thicknesses varying from 4 uc to 50 uc (Fig. 2a–e). Moreover, for K-dosed FeSe_0.93_S_0.07_ bulk crystals with no FeSe/oxide interface, a superconducting gap is also observed at 31 K (Fig. 2f). The gap size Δ is about 10 meV at 31 K for all the films and bulk FeSe_0.93_S_0.07_ (Fig. 2g). Comparing the temperature dependence of the gap size in the 30-uc and the 10-uc FeSe films as an example, the gaps are both 6 meV in size at 42 K (Fig. 2h), and close around 46 K (Fig. 2i,j). The temperature dependences of the gap sizes are summarized in Fig. 2k, in which all samples can be well fit by the same Bardeen–Cooper–Schrieffer formula with a *T*_c_ around 46 K. Therefore, for thick films or bulk material, the enhanced superconductivity here is intrinsic to the electron-doped FeSe, and is not dependent on the thickness or the FeSe/SrTiO_3_ interface, which is distinct from the previous report on K-dosed FeSe (ref. 24).

### Doping dependence

**Figure 3:**
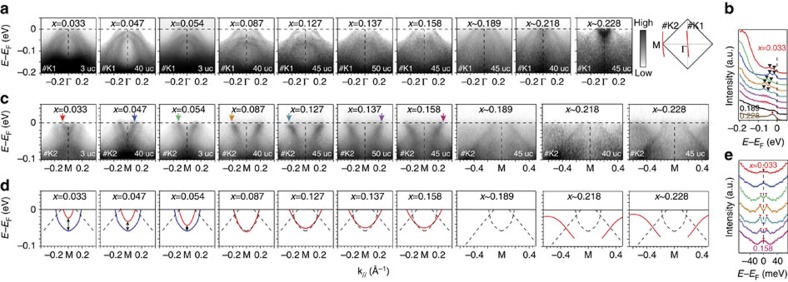
Evolution of electronic structure and superconducting gap as a function of electron doping induced by K dosing. (**a**) Evolution of photoemission spectra along cut #K1 in the inset as a function of increasing electron doping. The inset shows the Brillouin zone and the red curves indicate the momentum location of the cuts #K1 and #K2. (**b**) Energy distribution curves (EDCs) at Γ with different dopings. Triangles mark the band tops for the two parabolic bands after K dosing. For *x*=0.137 and *x*=0.158, only diffuse spectral weight can be observed as indicated by the pink shadow. For *x*∼0.189, the spectral weight could not be resolved. At *x*∼0.228, there is a well-defined peak near the Fermi energy, which is the band bottom of the electron-like band. (**c**) Doping-dependent evolution of photoemission spectra along cut #K2. (**d**) Doping-dependent evolution of the dispersions around M extracted from **c**. The solid curves indicate the electron-like bands of the doped surface layer, which are determined by parabolic fits to the dispersions in **c**. The dashed curves indicate the dispersions from undoped interior layers. (**e**) Symmetrized EDCs showing the evolution of the superconducting gap as a function of doping. The momenta of spectra are indicated by the arrows in **c** with corresponding colours. The data in this figure were taken at 31 K, except those for *x*=0.033 and *x*=0.054, which were taken at 25 K. The doping levels are calculated based on the Luttinger volume, with an uncertainty of ±0.005 electrons per Fe for *x*≤0.158 and ±0.01 electrons per Fe for *x*>0.158.

The evolution of the electronic structure with electron doping is further studied through ARPES on FeSe with systematically controlled K dosing. Figure 3a shows the spectra around Γ as a function of doping. For all the spectra at all doping levels, dispersions from the undoped interior FeSe layers are always visible, and do not depend on the doping at the surface. As the electron doping level *x* of the surface FeSe layer is increased from 0.033 to 0.127, the two hole-like bands gradually shift to higher binding energies (Fig. 3a,b). Simultaneously, these two bands become flat for *x* from 0.054 to 0.127, then become incoherent for *x*=0.137 and 0.158, and finally disappear for *x*∼0.189 (Fig. 3a), indicating increasing correlation strength with higher electron doping. As shown in Fig. 3b, the two quasiparticle peaks at Γ devolve into incoherent spectral weight (pink shadow in Fig. 3b) when *x*=0.137 and 0.158, and totally disappear once *x* reaches 0.189. On further doping to *x* ∼0.228, there is an electron-like band around the zone center (Fig. 3a), with well-defined quasiparticle peaks and no gap at 31 K (Fig. 3b, Supplementary Fig. 2). The electron-like band gradually sinks to higher binding energies as *x* increases from 0.218 to 0.232, and disperses distinctly from the quantum well states of potassium (Fig. 3a, Supplementary Fig. 3). A recent scanning tunnelling spectroscopy study has shown an unoccupied electron band in single-layer FeSe/SrTiO_3_ (ref. 35). The electron band observed in FeSe with *x* ∼0.228 could have the same origin, which is a partially occupied band of FeSe due to the heavy electron doping. These results suggest a metallic phase in the overdoped regime. The well-defined quasiparticle dispersion for *x* ∼0.228 suggests that the impurity scattering of K dosing is negligible, and the behaviour of the incoherent and diminishing spectral weight from *x*=0.137 to *x* ∼0.189 is intrinsic.

Around M, two electron-like bands are observed for the K-dosed FeSe with *x*=0.033 (Fig. 3c), which are illustrated by the solid curves in Fig. 3d. Compared with the undoped band structure in Fig. 1e, the upper band shifts downwards and the lower band remains at a fixed binding energy. Since the energy separation between them reflects the strength of the nematic order^26^, the decreased energy separation with increasing doping indicates the weakening of nematicity. As the doping further increases, the two electron bands become degenerate at the Fermi energy for *x*=0.087 and remarkably becomes flatter as *x* increases from 0.087 to 0.158, indicating enhanced correlations for bands around M, consistent with the behaviour of the bands around Γ. Remarkably, for *x* ∼0.189, the bands from the topmost layer becomes incoherent and the corresponding spectral weight is depleted at the Fermi energy. The depletion of spectral weight at the Fermi energy for the K-dosed bands around both Γ and M indicates that FeSe becomes insulating in this regime. On further electron doping to *x* ∼0.228, K-dosed FeSe shows dispersive bands below the Fermi energy. The band around M might be due to some folding and hybridization effects if certain charge or spin order exists in the insulating regime and persists to the metallic regime, which is a speculation and deserves further investigation. We emphasize that the data shown here were taken on four different samples with thicknesses of 3, 40, 45 and 50  uc (noted in Fig. 3a,c), and they have been reproduced in another six samples. The band dispersions evolve in the same manner, regardless of film thickness.

The symmetrized energy distribution curves in Fig. 3e give the doping dependence of the superconducting gap. The superconducting gap is observed at 25 K for the doping level 0.054, indicating a coexistance regime in which the superconductivity is enhanced while the nematicity is not fully suppressed. On the basis of empirical fitting of the superconducting gap^36^, the gap size increases to ∼9.7 meV at 31 K for films with *x*=0.087, and is slightly enhanced to 11.3 meV from *x*=0.087 to *x*=0.127, and then decreases to 7.7 meV at *x*=0.137, indicating an optimal doping around 0.127. The *T*_c_ increment for *x*≤0.12 in K-dosed FeSe is consistent with that in single-layer FeSe/SrTiO_3_ (ref. 7). The gap closes for *x*=0.158, suggesting that *T*_c_ falls below 31 K. The sample with *x* ∼0.228 is not superconducting at 31 K (Supplementary Fig. 2).

### Correlation effects and phase diagram

**Figure 4:**
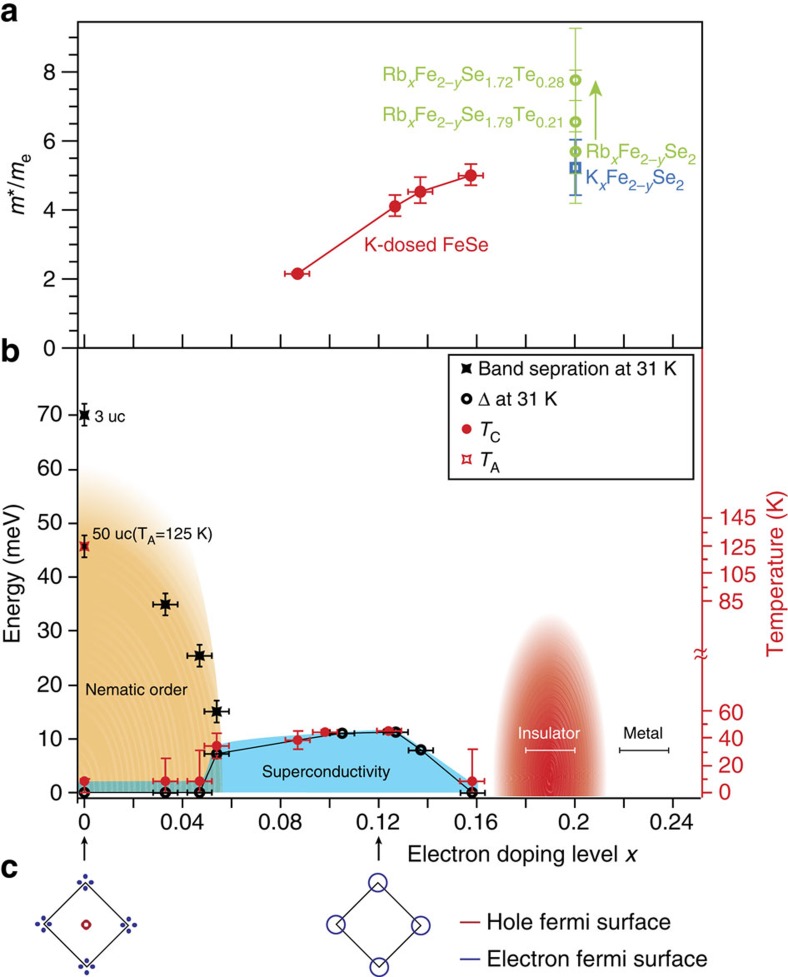
Phase diagram of FeSe as a function of electron doping. (**a**) Effective mass of the electron band at M as a function of doping; m_*e*_ is the mass of free electrons. The data points of K-dosed FeSe were obtained by the parabolic fits, and the error bars of the effective mass are due to the s.d. of the fitting process. The data points of Rb_*x*_Fe_2−*y*_Se_2_ and K_*x*_Fe_2−*y*_Se_2_ were from ref. 37. (**b**) Phase diagram of electron-doped FeSe, and the summary of the nematic band splitting, superconducting gap size and the *T*_c_ as a function of doping. The nematic band splitting was determined by the energy difference between band bottoms at M, while the undoped value is from ref. 6. For dopings without a superconducting gap at 31 K, the values of *T*_c_ were set at the *T*_c_ of bulk FeSe, 8 K. Otherwise, the values of *T*_c_ were determined by the superconducting gap-closing temperature. The gap sizes at 31 K were obtained through empirical fitting of the symmetrized energy distribution curves to a typical superconducting-state spectral function, and their error bars are due to the s.d. of the fitting process. The uncertainty in the electron doping is ±0.005 electrons per Fe for *x*≤0.158 and is ±0.01 electrons per Fe at higher dopings. Temperature error bars are due to measurement uncertainties. The energy error bars of the band splitting are due to the finite width of the spectra. Color gradients illustrate the uncertainty in the domain boundaries. (**c**) Different Fermi surface topologies of undoped FeSe and heavily electron-doped FeSe.

Figure 4a summarizes the effective mass of the electron band around M obtained from parabolic fits. The band mass increases monotonically in the doping range *x*=0.087–0.158 for K-dosed FeSe, while those of Rb_*x*_Fe_2−*y*_Se_2_ and K_*x*_Fe_2−*y*_Se_2_ at the electron doping level of 0.2 (ref. 37) follow the same trend, suggesting enhanced correlation strength with increasing electron doping.

Figure 4b shows the phase diagram of K-dosed FeSe as a function of doping. Because of experimental constraints, the superconducting gaps of the undoped and underdoped FeSe could not be determined, when the *T*_c_ is <25 K. However, it is known that the superconductivity coexists with nematic order in undoped FeSe crystal at low temperatures^27^,^29^,^30^. Our results extend the coexistence regime to *x*∼0.054, where *T*_c_ even reaches >25 K. By summarizing the superconducting gap size at 31 K, and the *T*_c_ determined by the gap-closing temperature (Supplementary Fig. 4), we obtain a superconducting dome with enhanced superconductivity near the nematic phase. The maximum *T*_c_ is ∼46 K, which is significantly enhanced compared with that in undoped FeSe. More intriguingly, an insulating phase eventually emerges, following a continuous increase of the effective mass with doping, suggesting that the insulating phase is driven by strong correlations. Further enhancement of the electron doping tunes the insulating state into a metallic phase with the Fermi crossings only around Γ, which has not previously been explored.

## Discussion

The phase diagram of the K-dosed FeSe has some of the essential ingredients of the canonical phase diagram of the iron-based superconductors. For example, the superconductivity is enhanced when the nematic order is suppressed, suggesting that the competition between nematicity and superconductivity likely plays an important role on the enhanced superconductivity^38^. The superconductivity is suppressed at higher dopings. Besides these essential ingredients, however, from an electronic structure perspective, the phase diagram is rather exotic and exhibits the following unique features.

First, superconductivity with a maximum *T*_c_ of 46 K is achieved in K-dosed FeSe for an optimal doping *x*∼0.12. The *T*_c_ in K-dosed FeSe is higher than the optimal *T*_c_ of 8 K in FeSe bulk crystals at ambient pressure^32^, that of 20 K in FeSe nanoparticles^39^, and that of 37 K in FeSe bulk crystals under high external pressure^40^. Indeed, it approaches the highest *T*_c_ in all heavily electron-doped FeSe-based bulk crystals, which is 48 K in *A*_*x*_Fe_2−*y*_Se_2_ under high pressure^41^. The high *T*_c_ in optimally K-dosed FeSe provides insight in understanding the origin of the high *T*_c_ in single-layer FeSe/SrTiO_3_, considering that they are both a single-unit-cell layer of FeSe having an electron doping *x*∼0.12 (refs 6, 7). On the basis of the pairing temperature measured by ARPES, the electron doping alone in an FeSe layer can enhance the *T*_c_ to 46 K, which is lower than the *T*_c_ of 65 K in single-layer FeSe/SrTiO_3_ determined in the same way^6^,^7^. Moreover, if considering the 109 K *T*_c_ found in the recent *in situ* transport measurements on single-layer FeSe/SrTiO_3_ (ref. 42), the interfacial effects beyond carrier doping could enhance *T*_c_ by >60 K.

Second, in the overdoped regime, most cuprates and iron-based superconductors become more Fermi liquid like as the correlation strength decreases^25^. Remarkably, in K-dosed FeSe, the correlation strength is enhanced with increasing electron doping, which is qualitatively different from the behaviour in iron arsenides. Such an enhancement of electron correlation strength with increased doping is quite anomalous, considering that the correlation strength usually decrease when doped away from 3*d*^5^ for Fe-based superconductors^43^,^44^,^45^. For example, the bandwidths of NaFe_1−*x*_Co_*x*_As and LiFe_1−*x*_Co_*x*_As increase with electron doping^25^. Besides, iron-based superconductors are generally considered to be moderately correlated materials with a metallic parent phase. However, FeSe layer-based superconductors are evidently in the vicinity of insulating phases. For example, non-stoichiometric FeSe with the chemical formula Fe_4_Se_5_ and 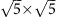 Fe vacancy order has been suggested to be a Mott insulator^46^, and while (Li,Fe)OHFeSe can be tuned into an insulating phase by enhancing the electron doping through liquid gating^47^. Here we have observed a superconductor-to-insulator transition in heavily electron-doped FeSe by K dosing. More importantly, the evolution to the insulating phase in K-dosed FeSe is characterized by the increasing effective mass and diminishing spectral weight of the coherent bands. Similar behaviour is observed through the metal-to-insulator transition of NiS_*x*_Se_2−*x*_ (ref. 48), which is considered to be a prototypical bandwidth-controlled Mott transition^49^. In the Brinkman-Rice picture, the quasiparticles become heavier until eventually their effective masses diverge in the insulating phase. Recently, such an enhancement of effective mass accompanied by a transition to an insulating phase has been observed in Rb_*x*_Fe_2−*y*_Se_2_ under chemical pressure^37^, which has the similar electron doping as the insulating phase in K-dosed FeSe (Fig. 4). Although a larger mass is observed before entering the insulating state for Rb_*x*_Fe_2−*y*_Se_2_ than that for K-dosed FeSe, these electron-doped iron chalcogenides with *x*∼ 0.2 probably share a similar correlation-driven superconductor-to-insulator transition route. We can speculate that there is likely certain magnetic and/or charge order in this insulating phase, which will need further investigation. Before entering the insulating phase, electron correlation is strengthened with higher K dosing, thus the spin susceptibility should be enhanced. Therefore, one possibility is that the spin susceptibility may diverge on approaching the insulating phase from lower dopings, and eventually an antiferromagnetic order may ultimately set in the insulating phase.

Third, an insulator to metal transition occurs on the far overdoped side. In this metallic phase, the Fermi surface consists of only a small electron pocket around Γ, while the bands near M sink below the Fermi energy. This Fermi surface topology is distinct from those of all other heavily electron-doped Fe-based superconductors, which consist of electron pockets near M. Although superconductivity is not observed at 31 K for this metallic phase, it remains to be explored at lower temperatures or at higher dopings.

Finally, this unique phase diagram connects two types of Fe-based superconductors with different Fermi surface topologies and different pairing symmetries. The Fermi surface of undoped FeSe consists of hole pockets at Γ and electron pockets around M (refs 6, 27, 28, 29, 30), while the superconducting paring symmetry is most likely s_±_ type with sign reversal between the hole and electron pockets (Fig. 4c), as evidenced by previous experiments^50^. On the other hand, the Fermi surface of electron-doped FeSe consists of only electron pockets, and the superconducting pairing symmetry is proposed to be different from the usual s_±_ type^51^,^52^,^53^,^54^,^55^,^56^,^57^,^58^, and has been suggested to be plain s-wave pairing without any sign change for FeSe/SrTiO_3_ from recent STM studies^59^.

To summarize, we have obtained enhanced superconductivity in thick FeSe films and FeSe_0.93_S_0.07_ bulk crystals by K dosing, indicating that the *T*_c_ can reach ∼46 K in a single-unit-cell layer FeSe by electron doping without any interfacial effect. K-dosed FeSe serves as a clean FeSe layer-based superconductor with well-controlled electron doping and weak impurity scattering. The different *T*_c_ in K-dosed FeSe and Co-doped FeSe suggests strong pair breaking of Co in heavily electron-doped FeSe. More importantly, we discover a systematic evolution of electronic correlations, and establish the extraordinary phase diagram of FeSe upon electron doping. A correlation-driven insulating phase and a metallic phase are uncovered at high doping levels. Our findings offer FeSe films as a prototypical system for understanding the interplay between different phases, such as the evolution between different pairing symmetries, the superconductor-to-insulator transition, and the coexistence of nematic order and superconductivity.

## Methods

### Growth of FeSe films and single crystals

The thick FeSe films were grown on TiO_2_-terminated Nb:SrTiO_3_ (001) substrates. FeSe films were co-deposited with the Se flux twenty times greater than the Fe flux, while the substrates were kept at 370 °C, and then post annealed at 410 °C in vacuum for 2.5 h and directly transferred into the ARPES chamber. The single crystals of FeSe_0.93_S_0.07_ (*T*_c_=9.7 K) were grown using the flux method^34^,^60^.

### ARPES measurements

ARPES data were taken under ultrahigh vacuum of 1.5 × 10^−11^ mbar, with a discharge lamp (21.2 eV He-I*α* light) and a Scienta R4000 electron analyzer. The energy resolution is 7 meV and the angular resolution is 0.3°. The sample growth/cleaving, K deposition and ARPES measurements were all conducted *in situ*.

### K-dosing experiments

Electron doping is induced by depositing K atoms with a commercial SAES alkali dispenser; the sample temperature was kept between 30–50 K when depositing K atoms. This low temperature reduces the mobility of the deposited atoms, and thus the K atoms simply transfer electrons to FeSe without affecting the stoichiometry of the FeSe surface. The doping levels <0.158 were determined by ARPES based on the Luttinger volume of Fermi surfaces. Correlating the estimated electron doping from the Luttinger volume with the K coverage calculated from deposition time and the flux of K measured by quartz crystal microbalance, we obtain a relationship between the two parameters, which we modelled by exponential function. The function was used to estimate the doping levels of K-dosed FeSe with *x*>0.158. The uncertainty in the electron doping for *x*≤0.158 is ±0.005 electrons per Fe, which is estimated by the combination of the momentum resolution of ARPES measurements and the uncertainty in determining the size of the electron pockets. The uncertainty in the electron doping for *x*>0.158 is estimated as ±0.01 electrons per Fe, from the combination of momentum resolution, the experimental uncertainty in determining the K coverage, and the uncertainty in the extrapolation required. We found doping levels >0.24 hard to achieve by K dosing.

## Additional information

**How to cite this article**: Wen, C. H. P. *et al.* Anomalous correlation effects and unique phase diagram of electron-doped FeSe revealed by photoemission spectroscopy. *Nat. Commun.* 7:10840 doi: 10.1038/ncomms10840 (2016).

## Supplementary Material

Supplementary Information Supplementary Figures 1-4 and Supplementary Reference
