# Supplementary material for: Anomalous correlation effects and unique phase diagram of electron-doped FeSe revealed by photoemission spectroscopy
Source: Nat Commun. 2016 Mar 8;7:10840. doi: 10.1038/ncomms10840 (PMC4786746; doi:10.1038/ncomms10840)
Supplement: Supplementary Information — Supplementary Figures 1-4 and Supplementary Reference [file ncomms10840-s1.pdf]

Supplementary Figures

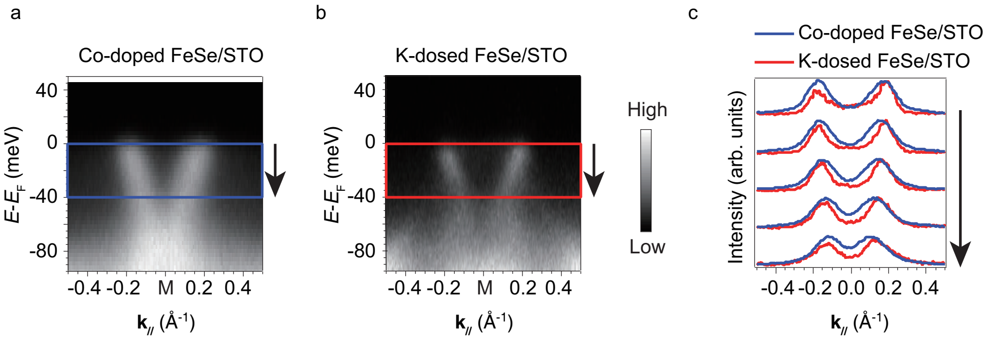

Supplementary Figure 1: **The different impurity scattering effects in Co-doped FeSe and in K-dosed FeSe.** (**a**, **b**) The photoemission spectra around M in Co-doped FeSe with the electron doping of 8% (ref. 1) taken at 30 K, and in K-dosed FeSe with the electron doping of 8.7% taken at 31 K, respectively. (**c**) The momentum distribution curves (MDCs) in the energy window shown in panels (a) and (b) for Co-doped FeSe and K-dosed FeSe, respectively. As shown in the main text, the K-dosed FeSe with 8.7% electron doping is superconducting above 31 K, however, the Co-doped FeSe with the electron doping of 8% is not superconducting at 30 K (ref. 1). Here the MDCs of the electron band in Co-doped FeSe are noticeably broader than those of K-dosed FeSe, indicating stronger impurity scattering due to Co substitution. The sharper lineshape of the momentum distribution curves and the enhanced superconductivity in K-dosed FeSe suggest the much weaker impurity scattering in FeSe doped by off-FeSe-plane K atoms.

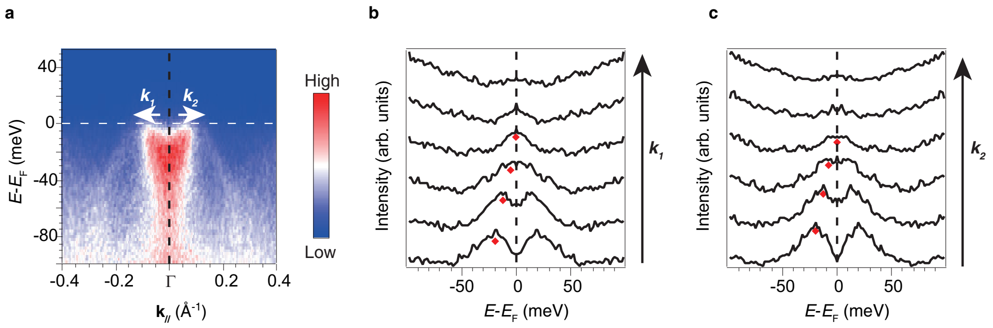

Supplementary Figure 2: **The metallic behavior of K-dosed FeSe with $x\sim$0.228 at 31 K.** (**a**) The photoemission spectra of the K-dosed FeSe films around Γ with $x\sim0.228$. An electron-like band is observed. (**b**, **c**) The symmetrized energy distribution curves with respect to the Fermi energy along $\boldsymbol{k}_1$ and $\boldsymbol{k}_2$, respectively; $\boldsymbol{k}_1$ and $\boldsymbol{k}_2$ are indicated by the white arrows in (a). The dispersions are marked by the red diamonds. The electron band disperses towards Fermi energy without any gap opening, indicating metallic behavior. The data were taken at 31 K.

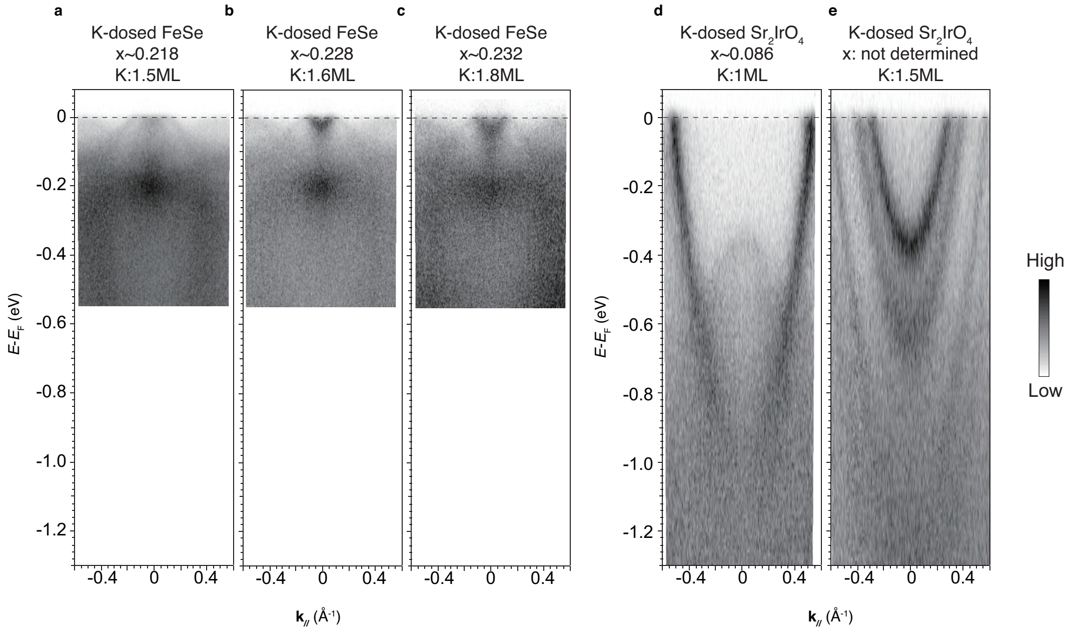

Supplementary Figure 3: **The evolution of the electron-like band in FeSe with *x* from 0.218 to 0.232 in the new metallic phase, and its comparison with that of the quantum well states of K.** (**a**- **c**) The electron-like band around Γ for K dosed FeSe with the doping of 0.218, 0.228, 0.232, respectively. An unoccupied electron band contributes some spectral weight at Γ for x~0.218. With further increased electron doping, there is an electron band visible at x~0.228, which sinks to higher binding energy for x~0.232. (**d**- **e**) The quantum well states of K observed in K-dosed Sr_2IrO_4. The surface coverage of K is shown in the figure; ML stands for monolayer. The electron band for K-dosed FeSe film shown in panel (a) shows dispersion very different from quantum well states of K observed in K-dosed Sr_2IrO_4 with the similar K coverage of 1.5ML shown in panel (e). Besides, as shown in panels (a)-(c), the K coverage is enhanced from 1.5ML to 1.8ML, while the electron doping in the topmost FeSe layer just varies from 0.218 to 0.232, indicating that there remains considerable electron density in the K overlayer. Based on the K coverage and the electron doping in the topmost layer, it can be estimated that K layer contributes about ~15% of its electron to the topmost FeSe layer, and about ~10% of its electron to the topmost Sr_2IrO_4 layer. The considerable electron density remaining in the K overlayer would induce quantum well states with large Fermi momenta as observed in K-dosed Sr_2IrO_4, which is not observed in K-dosed FeSe. Therefore, the electron band in the new metallic phase should not come from quantum well states of K. The absence of these states in K-dosed FeSe suggests a disordered arrangement of K atoms, perhaps in clusters rather than a uniform thin film.

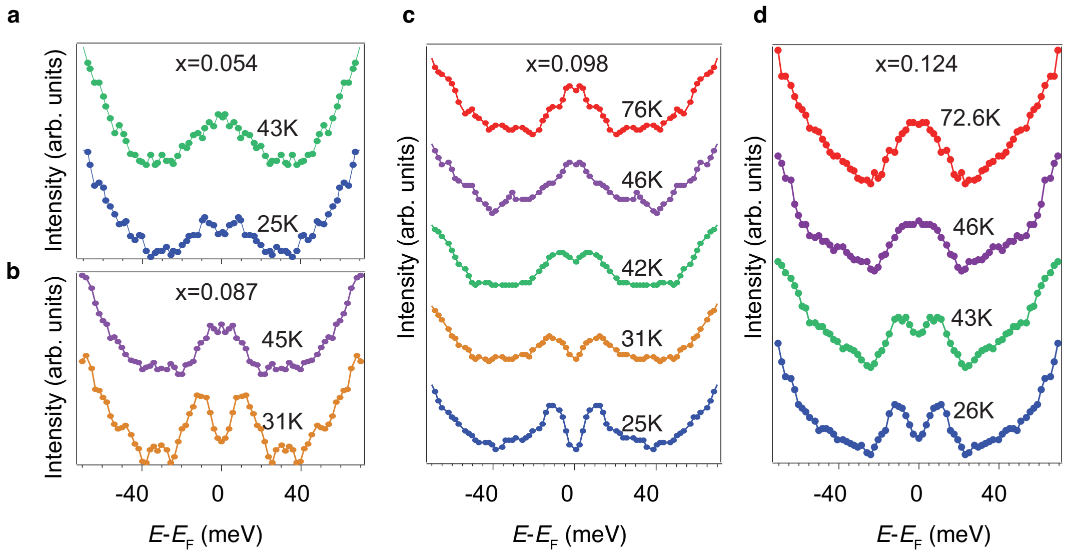

Supplementary Figure 4: **The temperature dependence of the superconducting gap for K-dosed FeSe with different electron doping.** (**a**) Temperature dependence of the superconducting gap for K-dosed FeSe with electron doping of x=0.054. (**b**) Temperature dependence of the superconducting gap for K-dosed FeSe with electron doping of x=0.087. (**c**) Temperature dependence of the superconducting gap for K-dosed FeSe with electron doping of x=0.098. (**d**) Temperature dependence of the superconducting gap for K-dosed FeSe with electron doping of x=0.124. For each film, a superconducting gap with sharp coherence peak presents a low temperature, decreases with increasing temperature, and eventually closes.

Supplementary Reference

[1] Peng, R. *et al*. Tuning the band structure and superconductivity in single-layer FeSe by interface engineering. *Nat. Comm.* **5**, 5044 (2014).
